# Hypoxia Signaling and Non-Coding RNAs: Regulatory Networks and Therapeutic Implications in Breast Cancer

**DOI:** 10.3390/cimb48010098

**Published:** 2026-01-18

**Authors:** Xin Hu, Rui Chen, Famin Ke, Dandan Wang, Xiaowei Gao, Can Song, Aimin Fu, Zuojin Ao, Hanyu Yang, Xiaoyan Liu, Xiurong Guo, Qiuyu Liu

**Affiliations:** 1 School of Pharmacy, Southwest Medical University, Luzhou 646000, Chinaswmuaozj@swmu.edu.cn (Z.A.);; 2 Laboratory of Metabolism, Center for Cancer Research, National Institutes of Health, Bethesda, MD 20892, USA

**Keywords:** breast cancer, hypoxia, hypoxia-inducible factor, non-coding RNA, molecular mechanism

## Abstract

The hypoxic microenvironment within breast cancer tumors leads to the sustained activation of hypoxia-inducible factors (HIFs), notably HIF-1α, which, in turn, triggers adaptive responses such as angiogenesis and metabolic reprogramming. These processes contribute to tumor invasion, progression, metastasis, and therapy resistance. Although a substantial portion of the human genome is transcribed into non-coding RNAs (ncRNAs), which have been shown to play key regulatory roles in the development and progression of breast cancer, the interplay between HIFs and ncRNAs—and how such crosstalk influences breast cancer pathogenesis—remains poorly understood. This review aims to systematically outline the mechanisms of hypoxia-related signaling and ncRNA function in breast cancer, with a focus on their molecular interactions in disease progression and their potential clinical implications.

## 1. Introduction

Breast cancer is among the most common malignant tumors in women, which develops in the epithelial tissue of the breast. Although its exact cause is unknown, a number of factors, including genetics (BRCA1/BRCA2 gene mutations), hormone levels (long-term stimulation by progesterone and estrogen), and lifestyle (high-fat diet), may be involved. Hypoxia is typically observed in the microenvironment of breast cancer tumors, leading to the overexpression and activation of hypoxia-inducible factors (HIFs) in breast cancer [1]. The HIF family plays a crucial role in the intricate relationships between breast cancer tumor cells and stromal cells. HIF can cause angiogenesis, metastasis, and metabolic reprogramming under hypoxic settings by controlling the expression of downstream pathway genes [2,3], greatly accelerating the growth of tumors [4]. Additionally, pertinent findings indicate a substantial association between a poor prognosis for breast cancer and its expression level. As a key molecular hub for regulating cancer development in hypoxic microenvironments, HIFs participate in the entire process of breast cancer development by multidimensional regulation of the hypoxia-inducible signaling pathway.

Non-coding RNAs (ncRNAs) are implicated in human malignant tumors, according to a substantial body of research [5]. They can regulate the basic biological processes of tumor cells by functioning as oncogenes or tumor suppressor genes, such as invasion, apoptosis, and proliferation [6]. Numerous ncRNAs that serve as prognostic or diagnostic markers can be discharged from cancer cells into the blood or urine [7]. Nevertheless, the non-coding regulatory mechanism of hypoxia in breast cancer remains unclear, particularly the bidirectional regulatory network between HIFs and ncRNAs. The distinct action patterns of ncRNA types in different subtypes of breast cancer have not been thoroughly and methodically explained and analyzed in terms of clinical relevance, lacking conclusive findings [8]. Thus, we summarized the bidirectional feedback regulatory network connecting miRNA, lncRNA, circRNA, piRNA, and HIFs, mainly different ncRNAs exhibiting distinct expression patterns in various molecular subtypes of breast cancer. HIFs can induce the expression of particular ncRNAs, and vice versa, ncRNAs can control the activity and stability of HIF through ceRNA. Hypoxia-responsive ncRNAs can influence the course of breast cancer by controlling the tumor microenvironment and mediating treatment resistance. It has been demonstrated that some ncRNAs may be used as prognostic markers for breast cancer, and the creation of drugs that specifically target these ncRNAs has promise for improving clinical outcomes [9]. The specific regulatory roles of HIF-2α and HIF-3α in breast cancer are also briefly discussed in this review, which highlights how hypoxia activates downstream signaling pathways, creating a pro-cancer loop with the HIF–ncRNA regulation network. Their possible distinct molecular mechanisms encourage invasion and metastasis of breast cancer cells, hastening the progression of breast cancer, open a door of new possibilities for biomarkers in the assessment of breast cancer prognosis, offer a theoretical basis for the development of targeted therapeutic strategies that target the hypoxia–ncRNA regulatory network, and provide potential intervention concepts to address the problem of treatment resistance in breast cancer and enhance patient outcomes.

## 2. Breast Cancer

With its incidence and fatality rates steadily rising, breast cancer has become one of the primary malignant tumors endangering women’s health globally in recent years [10]. In terms of cancer incidence, female patients had the second-highest incidence rate of breast cancer, at roughly 51.17% of the population of China. Tumor cells go through a number of adaptive changes to increase their invasive and metastatic potential, including metabolic reprogramming, gene expression regulation, and signal pathway activation in a low-oxygen microenvironment because cancer cells grow more quickly. Surgery, chemotherapy, radiation, targeted therapy, and endocrine therapy are currently the primary treatments for breast cancer, and medication therapy has varying effects depending on various cancer subtypes. Usually, breast cancer can be divided into luminal (luminal A and luminal B), triple negative, and HER-2 positive based on the expression of estrogen receptor (ER), progesterone (PR), and human epidermal growth factor receptor 2 (HER2). The majority of breast solid tumors have a poor clinical prognosis due to their hypoxic microenvironment. Further evidence of the function of hypoxia in the early evolution of malignancies comes from reports that 40% of breast tumors and 50% of locally progressed breast cancers have hypoxic patches at the time of diagnosis [11,12]. Intratumoral hypoxia increases metastasis and lowers survival rates in breast cancer patients [13]. Tumor growth is limited to 1–2 mm because the oxygen diffusion limit is 180 μm; otherwise, the tumor will become hypoxic [14]. When the tumor grows larger than this, HIFs trigger a number of intricate stress reactions that impact the proliferation of cancer cells, such as the activation of angiogenesis, cell growth and survival, invasion and metastasis, glucose metabolism, and immune system evasion.

## 3. Hypoxia

The body’s regular physiological states are impacted by hypoxia. Tumor hypoxia is caused by a supply–demand imbalance brought on by the fast growth of tumor cells and the sluggish and disorganized structure of angiogenesis. Hypoxia has a variety of effects on cancer, including encouraging its growth and decreasing the efficacy of treatment. Clinically, the prognosis of patients is strongly correlated with the level of hypoxia in malignancies. Tumors with significant hypoxia typically show greater invasive and metastatic potential in addition to being less responsive to radiation, chemotherapy, and immunotherapy. As a result, targeted hypoxia treatment has emerged as a key focus of cancer research. Statistics show that hypoxic regions accompany 50–60% of solid tumors, and HIFs are essential for tumors to adapt to hypoxic conditions [15,16]. Hypoxia, one of the main characteristics of the solid tumor microenvironment, can trigger the HIFs and start the adaptive regulatory system in tumor cells. The HIF family plays a crucial hub function in linking hypoxia signals with physiological changes in tumors because HIFs are the primary transcription factors that detect changes in oxygen concentration and trigger adaptive responses. The intricacy and variety of hypoxia adaption mechanisms are directly determined by the composition structure and functional variations in its constituents.

### 3.1. Hypoxia and HIFs

Hypoxia induces the activation of the HIFs, triggering the coordinated action of multiple key intracellular signaling pathways. HIF-1α, HIF-2α, and HIF-3α are the three different members of the heterodimeric basic-helix-loop-helix-PAS transcription factors that make up the HIFs transcription factor family. Oxygen primarily regulates the activity of HIFs through the α subunit, which is both an active and regulatory subunit of HIFs. While the HIF-1α subunit has been the primary focus of research, recent studies have discovered that HIF-2α, which differs from HIF-1 [17], can form heterodimers with HIF-1β and contribute to the regulation of hypoxia response genes under chronic hypoxia circumstances [18,19]. Despite having 48% amino acid sequence identity, HIF-1α and HIF-2α have diverse functions and gene targets [17,20], and their roles change in various cancers and cell types. The lungs, heart, placenta, adult blood vessels, and embryonic cells all have large amounts of HIF-2α [19]. HIF-3α has a distinct amino acid sequence when compared to HIF-1α and HIF-2α, sharing 57% and 53% amino acid sequence identity with HIF-1α and HIF-2α in the bHLH-PAS domain and 61% homology with HIF-1α in the oxygen-dependent degradation domain (ODD) [18]. The target genes of HIF-1α and HIF-2α are thought to be negatively regulated by HIF-3α [21], and the HIF-1α subunit, in particular, is essential for the development of cancer.

The typical HIF-α subunit (primarily HIF-1α) binds to the von Hippel–Lindau (VHL) protein under normoxic conditions, activating the ubiquitin ligase system and making it vulnerable to degradation by the proteasome complex. The classic hypoxia molecular signaling pathway is centered on HIFs, which control the adaptive response of cells to oxygen homeostasis imbalance. Prolyl hydroxylase domain (PHD) enzymes, which catalyze the proline hydroxylation of HIF-α after identifying oxygen molecules, are the main cause of this process [22]. The VHL protein complex then recognizes HIF-α and mediates its ubiquitination and degradation, which is dependent on several hydroxylases, including α-ketoglutarate-dependent dioxygenases and prolyl hydroxylases. The intracellular asparagine hydroxylation factor hydroxylates the asparagine residue of HIF-α, preventing it from binding to the co-activator p300 and then reducing HIFs’ transcriptional activity [23,24]. Under hypoxic conditions, HIF-α is prevented from breaking down and dimerizes with HIF-1β to create a subunit dimer. GLUT-1, VEGF, erythropoietin (EPO), and LDH-A are among the genes whose transcription and translation are regulated by the subsequent binding of dimers to the hypoxia response elements (HRE) of downstream proteins or enzymes [13]. HIF can contribute to the development of tumors and the adaptation of cells to hypoxia by multifaceted control of target genes. HIF stimulates the production of genes related to angiogenesis, such as vascular endothelial growth factor (VEGF), which, in turn, stimulates angiogenesis and supplies tumors with blood [18,25]. It causes the production of enzymes associated with glycolysis, which enables cells to produce energy through anaerobic glucose metabolism in hypoxic conditions. HIF primarily stimulates the synthesis of EPO, a hormone that encourages the development of red blood cells. Furthermore, HIF balances energy generation and consumption to aid cells in adapting to hypoxia in addition to boosting oxygen delivery, cell survival, and metabolism. Immunological cell function, cytokine generation, and other inflammatory-related activities are all impacted by HIFs’ involvement in controlling immunological responses and inflammatory processes under hypoxic settings.

**Figure 1:**
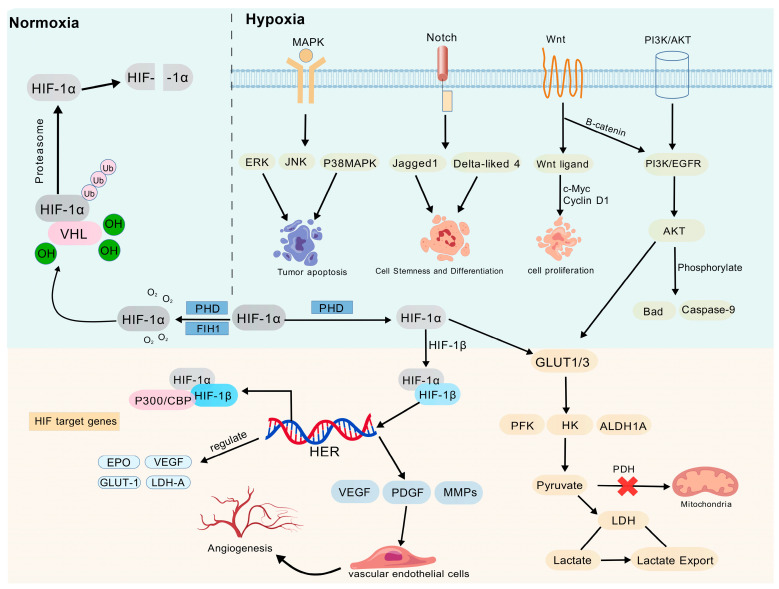
Regulation of HIF-1α and downstream signaling pathways under normoxic/hypoxic conditions. Under normoxic conditions, HIF-1α is hydrolyzed by prolyl hydroxylase domain (PHD). The von Hippel–Lindau protein (VHL) then recognizes and binds to PHD, causing the proteasome to degrade HIF-1α quickly. Under hypoxic conditions, HIF-1α translocates to the nucleus instead of degradation, where it binds to the hypoxia response elements (HRE) and forms a dimer with HIF-1β to regulate target genes. Through multifaceted control of target genes, HIF-1α also contributes to the development of tumors and the adaptation of cells to hypoxia. Hypoxia can also activate MAPK, Notch, Wnt, PI3K/AKT, and other signaling pathways to regulate tumor apoptosis, cell stemness and differentiation, cell proliferation, cell metabolism, and angiogenesis. HIF-1α—Hypoxia-Inducible Factor-1α; HIF-1β—Hypoxia-Inducible Factor-1β; VHL—Von Hippel–Lindau; PHD—Prolyl Hydroxylase Domain-containing protein; MAPK—Mitogen-Activated Protein Kinase; PI3K/AKT—Phosphatidylinositol 3-Kinase/Protein Kinase B; ERK—Extracellular Signal-Regulated Kinase; JNK—c-Jun N-Terminal Kinase; p38MAPK—p38 Mitogen-Activated Protein Kinase; EGFR—Epidermal Growth Factor Receptor; GLUT1/3—Glucose Transporter 1/3; PFK—Phosphofructokinase; HK—Hexokinase; ALDH1A—Aldehyde Dehydrogenase 1A; LDH—Lactate Dehydrogenase; EPO—Erythropoietin; VEGF—Vascular Endothelial Growth Factor; PDGF—Platelet-Derived Growth Factor; MMPs—Matrix Metalloproteinases; P300/CBP—E1A Binding Protein p300/CREB-Binding Protein; HER—Human Epidermal Growth Factor Receptor. Created with BioGDP.com accessed on 22 December 2025. Agreement number: GDP2025KJJ02H [27].

In tumor cells, hypoxia can quickly activate the PI3K/AKT, MAPK, Wnt, Notch, and other signaling pathways. Of these, the PI3K/AKT pathway is most strongly activated: HIF increases PI3K expression or activates upstream molecules like EGFR, which promotes PI3K phosphorylation and AKT activation. A “hypoxia-PI3K/AKT-HIF”-positive feedback loop is created when active AKT phosphorylates Bad and Caspase-9 to prevent apoptosis, increases the expression of GLUT and LDH to improve glycolysis, and keeps HIF stable by controlling its post-translational modification. Histone lactylation-mediated USP39 expression regulation has been shown to further amplify this loop in endometrial cancer and other malignancies, encouraging the malignant growth of tumors. Hypoxia activates the ERK, JNK, and p38 MAPK subtypes in the MAPK pathway. These subtypes regulate tumor cell proliferation, invasion, apoptosis, and autophagy balance, respectively, and promote malignant progression by activating the HIF-1α pathway through regulation of the P300/CBP protein complex. The Wnt pathway is activated by upregulating Wnt ligands and preventing the degradation of β-catenin through target genes including c-Myc and Cyclin D1, preserving cell proliferation and stemness [26]. β-catenin can control HIF-1α function via PI3K/Akt signaling, while hypoxia-induced Jagged1, Delta-like 4 (DLL4), and other ligand expressions activate the Notch pathway. DLL4 is a key ligand that can activate Notch signaling through the HIF-1α-mediated regulatory pathway, regulating cell stemness and differentiation and forming a positive regulation with the HIF pathway to synergistically promote the formation of abnormal vascular networks and tumor invasion and metastasis, which has been confirmed in malignancies, including lung and colorectal cancer (Figure 1).

### 3.2. HIF in Breast Cancer

As stated in the previous section, HIF-1α activity in breast cancer dramatically increases under hypoxic settings. However, HIF-1α is unstable, and its activity declines under normoxic circumstances [28]. HIF-1α is an overexpressed isoform in breast cancer [29,30], and this characteristic has been clearly identified in precancerous lesions like ductal carcinoma in situ (DCIS) and early-stage breast cancer. It mostly acts in the hypoxic core of the tumor and around necrotic foci. Tumor grade and invasiveness are strongly correlated with its expression level [31]. In addition to HIF-1α, research has shown that HIF-2α, which is controlled by upstream regulator FOXA1, plays a distinct function in the development and angiogenesis of breast cancer [32,33]. Numerous molecular mechanisms, including the loss of tumor suppressors PTEN, p53, or BRCA1, as well as the overactivation of the PI3K/Akt/mTOR or MAPK pathways, regulate the abnormal activation of HIF-1α and HIF-2α in breast cancer. These mechanisms can increase the transcription, translation, or stability of HIF-α. About 70% of breast cancers are ERα positive, while 15% to 30% of human breast tumors exhibit overexpression of HER-2 in clinical subgroups, and both of these factors raise HIF-α levels by boosting PI3K/Akt/mTOR signaling [34,35]. ERα can directly stimulate the production of HIF-1α instead of HIF-2α through the estrogen response element in the HIF-1α promoter [4,35,36,37,38].

**Figure 2:**
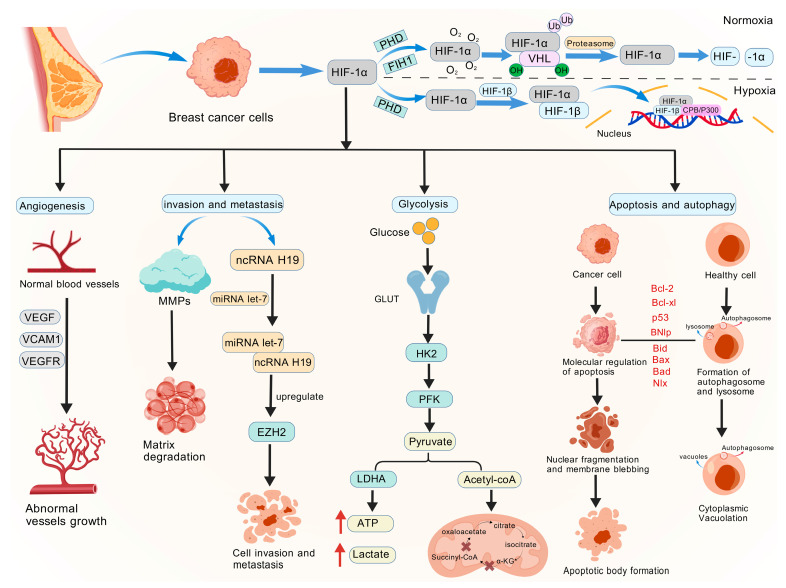
The role of HIFs in breast cancer. Under normoxic conditions, HIF-1α is unstable and has a short half-life. Under hypoxic conditions, HIF-1α translocates to the nucleus, where it forms a dimer with HIF-1β, further participating in angiogenesis, glucose metabolism, cancer cell invasion and metastasis, autophagy, and apoptosis by regulating target genes. HIF-1α—Hypoxia-Inducible Factor-1α; HIF-1β—Hypoxia-Inducible Factor-1β; PHD—Prolyl Hydroxylase Domain-containing protein; VHL—Von Hippel–Lindau; ncRNA—Non-Coding RNA; miRNA—MicroRNA; EZH2—Enhancer of Zeste Homolog 2; GLUT—Glucose Transporter; HK2—Hexokinase 2; PFK—Phosphofructokinase; LDHA—Lactate Dehydrogenase A; Acetyl-CoA—Acetyl Coenzyme A; Succinyl-CoA—Succinyl-Coenzyme A; MMPs—Matrix Metalloproteinases; VEGF—Vascular Endothelial Growth Factor; VEGFR—Vascular Endothelial Growth Factor Receptor; VCAM-1—Vascular Cell Adhesion Molecule 1; Bcl-2—B-cell Lymphoma 2; p53—Tumor Protein p53; Bax—BCL2-Associated X Protein; BNIp—BCL2/adenovirus E1B 19 kDa-interacting protein; Bid—BH3-interacting domain death agonist; Bad—BH3-domain agonist; NIx—BCL2/Adenovirus E1B 19 kDa protein-interacting protein 3-like; Bcl-xl—B-cell lymphoma-extra large. Created with BioGDP.com accessed on 22 December 2025. Agreement number: GDP20259JFJWW [27].

Hypoxia and HIF pathway activation are the main factors that control the biological activity of breast cancer in the following different ways. Initially, it promotes the growth of tumor neovascularization. When HIF-1α is activated, VEGF, which provides nutrition and oxygen to rapidly proliferating breast cancer cells, is increased. The circumstances for further metastases are created by the increased permeability of newly generated blood vessels. It is a crucial stage in the development of invasive carcinoma from carcinoma in situ, and there is a significant correlation between the density of tumor microvessels and the expression level of HIF-1α. Moreover, it increases the capacity of breast cancer cells to invade and spread. HIF-1α not only accelerates the breakdown of the extracellular matrix but also directly regulates the expression of invasion-related genes such as matrix metalloproteinases (MMPs) through the ncRNA network. For instance, lncRNA H19, which acts as a competitive endogenous RNA (ceRNA) to compete with EZH2 mRNA for binding to miRNA let-7, can be expressed more when there is hypoxia [39]. It indirectly increases the expression of EZH2 and encourages the invasion and metastasis of breast cancer cells [40,41]. In addition, it can promote the epithelial–mesenchymal transition (EMT) process and alter the cytoskeleton, which raises the risk of tumor spread. Furthermore, it encourages the emergence of metabolic reprogramming and medication resistance. The HIF pathway may induce breast cancer cells to switch from aerobic respiration to glycolysis by boosting the expression of crucial glycolytic enzymes, including glucose transporter (GLUT) and hexokinase (HK), ensuring an energy source in hypoxic conditions (1). By simultaneously doubling the quantity of lipid particles and leveraging energy storage mechanisms to counteract drug-induced assaults, the synergistic interplay of hypoxia and hypercapnia further suppresses the anticancer activity of immune cells while enhancing chemoresistance in tumor cells, thereby markedly increasing tumor cell survival rates (Figure 2).

HIF-1α is consistently strongly expressed in triple-negative breast cancer (TNBC), with a poor prognosis [42,43,44]. The 18F-fluoromisonidazole PET tracer, which builds up preferentially in hypoxic cells, was well absorbed by TNBC patients [45]. Even in normoxic conditions, TNBC cells exhibit hypoxia gene signatures [46]. PTEN mutation, EGFR overexpression, and p53 deletion are common changes in TNBC that may lead to elevated HIF activity, and the HIF response in TNBC may be controlled by transcription factor X-box binding protein 1 [47,48]. PTEN and p53 mutations are well-documented to contribute to elevated HIF activity in breast cancer. Notably, the regulatory pattern of HIF exhibits specificity in triple-negative breast cancer (TNBC): its activity enhancement is mainly achieved through the modulation of protein stability rather than mRNA transcription. Interestingly, enhanced HIF-α mRNA levels were not found in TNBC cells, indicating that significant post-transcriptional mechanisms play a role in high HIF activity [46]. Additionally, intracellular cysteine depletion is related to PHD malfunction and paracrine glutamate signaling and can stabilize HIF-1α in TNBC under normoxic circumstances [42]. Other metabolites and HIF-induced metabolic enzymes that generate positive feedback loops with HIF activity include reactive oxygen species (ROS), acetyl-CoA synthetase 2 (ACSS2), and mitochondrial proteins like CHCHD [49]. The expression, stability, and effector function of HIF-α on HRE can be affected by bidirectional processes such as circadian rhythms, ncRNAs, HIF-dependent microvesicle secretion by tumor cells or cells in the tumor microenvironment, and epigenetics impact [50,51,52,53,54].

Heat shock proteins (HSP) can regulate metabolic reprogramming, which is a crucial tactic used by breast cancer cells to adapt to hypoxic settings. HIF-1α facilitates the change in cellular energy metabolism under hypoxic conditions by upregulating glycolytic enzymes and redistributing pyruvate to produce lactate through multiple pathways [55]. Lactate in breast cancer tumor cells can cause the production of programmed death ligand (PD-L1), which allows tumor-specific antigens to elude immune cells and accelerate tumor growth [56]. Increased exposure to lactate can influence MCF-7 cells’ phenotype and increase tamoxifen resistance, and high lactate levels may worsen clinical outcomes by improving the characteristics of cancer stem cells in individuals with breast cancer [57]. Hypoxia can result in lactate synthesis and release, which can acidify the extracellular microenvironment [58], which strengthens the chemoresistance of breast cancer cells by increasing glycogen production and glucose absorption [59]. Tumor immune suppression in breast cancer may result from the buildup of extracellular lactate and the acidification that follows. MCT-1 and MCT-4 and hypoxia-induced carbonic anhydrase 9 are essential in this process [2,60]. While MCT-4 encourages the efflux of lactate and hydrogen ions in breast cancer cells, MCT-1 catalyzes the transformation of carbon dioxide and water into bicarbonate ions and hydrogen ions [2,3,55,58,59,60,61,62,63,64,65,66]. Hypoxic cells require extra supplies of tricarboxylic acid cycle intermediates, such as cysteine and glutamine, as the majority of pyruvate in tumor cells is diverted away from the cycle. It is accomplished by upregulating a number of amino acid transporters that are HIF response genes [57], including SNAT2, SLC1A5, ASCT2, SLC7A11, and SLC7A5 [67]. Simultaneously, elevated activity of glutaminase transforms glutamine into glutamate [68]. Fatty acids and lipids are essential for vital biological processes in cancer cells, including metabolism, signal transmission, intracellular oxidative adaption, and proliferation [69]. HIF-α proteins can prevent fatty acid oxidation and increase the synthesis of fatty acids by transcriptionally activating enzymes such as fatty acid synthase, Lipin-1, and acetyl-CoA carboxylase (ACC), while HIF-α can dramatically increase the absorption of fatty acids by upregulating fatty acid-binding proteins [70].

Recent research has shown that HSPs can modify the methylation state of DNA and nuclear histones to govern epigenetic programs in addition to temporary transcriptional responses [52]. As histone lysine demethylases (KDMs) maintain the HIF-1α complex and start the transcription of its important target genes, their activation by HSPs is essential in this process. KDMs belong to the 2-oxoglutarate-dependent dioxygenase family (KDM3A, KDM2B, KDM4B, KDM5B, KDM6B, and KDM4C), and their enzymatic activity depends on oxygen and 2-oxoglutarate [52]. These enzymes are crucial agents for controlling chromatin structure in addition to acting as signal amplifiers and transcriptional activators of downstream genes in the HIF pathway [71].

HSPs can provide a long-lasting response to hypoxia via the epigenetic pathway, and hypoxia can reduce TET demethylase activity, leading to DNA hypermethylation [72]. Actually, it is possible to transcribe a large number of DNA sequences that are thought to be worthless yet have significant biological activities [73,74,75]. NcRNA genes have been demonstrated to be an important source of recently discovered regulatory elements connected to a spectrum of physiological and pathological conditions, particularly in cancer, due to their complex and deep genomic rearrangement properties [76].

## 4. NcRNAs

RNA makes up 70% of human DNA, although less than 2% of it codes for proteins [67,77]. The genomes of prokaryotes and eukaryotes contain a large amount of ncRNA, a form of RNA molecule with significant biological roles that does not encode proteins. For a very long period, ncRNA was thought of as the “dark matter” or “junk sequence” in the genome, and its roles remained unclear. It has been found that a kind of ncRNA that was once thought to be transcriptional noise is crucial to daily operations. Numerous researchers have already identified a significant number of ncRNAs using high-throughput sequencing, bioinformatics, and other techniques [78]. In this section, we have compiled ncRNAs associated with various breast cancer subtypes, as summarized in Table 1. NcRNAs are categorized according to their location, length, and form: microRNAs (miRNAs), long non-coding RNAs (lncRNAs), circular RNAs (circRNAs), and PIWI-interacting RNAs (piRNAs) are the four primary types of ncRNAs with distinct roles in cancer [7]. Target mRNAs can be degraded by RNA-induced silencing complexes (RISCs) when miRNAs attach to their complementary sequence [79]. PiRNAs were first discovered in fruit flies, and their length is 24–30 nucleotides, mostly presenting in germ cells and interacting with PIWI family proteins to participate in chromatin epigenetic regulation [80]. LncRNAs and circRNAs are longer than 200 nucleotides, and their morphology is the primary distinction between them: circRNAs are circular, whereas lncRNAs are linear. Both lncRNAs and circRNAs can fold into intricate secondary structures that enable their interactions with DNA, RNA, and proteins, transcribed from exons, introns, intergenic regions, or 5′/3′-untranslated regions [81]. CircRNAs and lncRNAs control gene expression in a variety of ways, serving as miRNA decoys to stop target mRNAs from being degraded and functioning as scaffolds to govern protein–protein interactions and associated downstream signaling cascades in addition to controlling the binding of transcription factors to promoters to regulate the expression of target genes.

### 4.1. NcRNAs and Cancers

One benefit of ncRNAs is their ability to control gene expression. Small ncRNAs (19–24 nt, miRNAs) are where this starts. These microstructures are transcribed from the genome as primary miRNAs, which are subsequently cleaved into precursor miRNAs (pre-miRNAs) in the nucleus by molecules including DROSHA and DGCR8. Exportin 5 (XPO5) carries the pre-miRNAs to the cytoplasm, where TRBP and Dicer enzymes help cleave them into mature miRNAs. After binding to the 3′ untranslated region (3′ UTR) of mRNAs, these mature miRNAs create miRNA-mediated silencing complexes (RISC), which impede or degrade mRNA translation [98,99]. Mammals contain a large number of miRNAs, which are significant molecules involved in many aspects of life. MiRNAs can maintain intracellular homeostasis by attaching to and blocking proteins, such as blocking nuclear ncRNAs, blocking mitochondrial transcripts, encoding peptides, starting transcription [100], triggering mRNA translation, and activating Toll-like receptors [101].

LncRNAs, a class of regulatory ncRNAs, are made up of RNA molecules with about 200 nucleotides. They are functioning RNA molecules that cannot encode whole proteins because they lack effective open reading frames. LncRNAs are found all around the genome, and they are processed and transcribed similarly to mRNAs. Nonetheless, they frequently create flexible, multifaceted three-dimensional structures that can alter quickly and serve a variety of purposes [102]. RNA polymerase II is primarily responsible for their transcription [103]. A class of ncRNAs with a variety of roles is known as lncRNAs, exhibiting little sequence conservation across species and high tissue and cell selectivity [104]. However, they play a significant role in the multi-level control of gene expression and can produce specific functional domains via secondary or tertiary structures [104]. They can also operate as signal molecules, molecular scaffolds, targeting guides, regulatory decoys, etc. [105]. In addition, these proteins can modify chromatin, loop chromosomes, transcribe genes, bind to proteins and mRNAs, interact with other ncRNAs, translate proteins, and generate extranuclear bodies. For example, miRNAs and lncRNAs can act as tumor suppressor or oncogene genes, controlling the growth and occurrence of cancers.

CircRNA, a kind of ncRNA molecule that is frequently found in eukaryotic cells and forms a circular structure by joining the ends, is the third type of ncRNA having regulatory roles. Complex biological processes like exon skipping, intron lariat creation, intron pairing, and RNA-binding protein dimerization are necessary for their production. CircRNAs can perform biological functions more steadily than linear RNA based on their circular structure since they are more resistant to nucleases and have a longer half-life in cells [106], which is in contrast to the 5′ cap and 3′ polyadenylated tail structure of conventional linear RNA. CircRNAs can attach to proteins, control protein translation, and adsorb miRNAs in order to preserve intracellular homeostasis [107].

**Figure 3:**
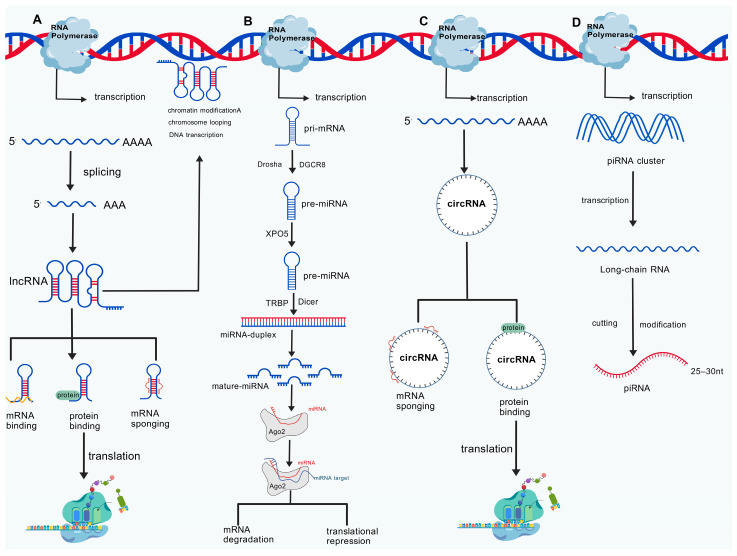
Transcriptional processing and regulatory functions of ncRNAs. (**A**) LncRNAs mature in the nucleus and are spliced to create intricate three-dimensional structures. They control DNA transcription, chromosomal looping, chromatin modification, and nuclear speckle formation outside of the nucleolus. LncRNAs can be carried to the cytoplasm, where they interact with proteins, messenger RNAs, and miRNAs to control the translation and modification of proteins. (**B**) MiRNAs are transcribed by RNA polymerase II as pri-miRNAs. After processing by the Drosha complex, pre-miRNAs are transported to the cytoplasm by XPO5. Mature miRNAs are generated through processing mediated by Dicer and TARBP2. MiRNAs function by degrading mRNAs or inhibiting translation, thereby regulating cancer. (**C**) CircRNAs mature through various biogenesis mechanisms, such as exon skipping, intron debranching, intron pairing, and RNA-binding protein dimerization. It can control the translation and function of proteins by directly interacting with and attaching to miRNAs. (**D**) PiRNAs are long RNAs transcribed from specific piRNA clusters in the genome. These precursor RNAs undergo a series of cleavages and modifications (such as 2′-O-methylation at the 3′ end) to form mature piRNA molecules. piRNAs silence transposons, regulate epigenetics, and participate in the cell cycle and invasion processes of breast cancer. mRNA—Messenger RNA; pre-mRNA—Precursor Messenger RNA; circRNA—Circular RNA; lncRNA—Long Non-Coding RNA; miRNA—MicroRNA; piRNA—Piwi-Interacting RNA; snRNA—Small Nuclear RNA; snRNP—Small Nuclear Ribonucleoprotein; RBP—RNA-Binding Protein; TRBP—TAR RNA-Binding Protein; XPO5—Exportin-5. Created with BioGDP.com accessed on 18 January 2026. Agreement number: GDP20261JJ020 [27].

PiRNA is primarily found in animal germ cells. Its primary characteristic is its specialized interaction with PIWI subfamily proteins of the Argonaute protein family to produce biologically useful piRNA/PIWI complexes. Unlike miRNA and siRNA, piRNA is produced via a Dicer-independent mechanism. Its precursors are long-chain RNAs that are transcribed from particular piRNA clusters in the genome, which are primarily repetitive sequence areas like transposon sequences. These precursor RNAs go through a number of cleavages and modifications before becoming mature piRNA molecules, including 2′-O-methylation at the 3′ end, and these changes can increase their stability and stop nuclease degradation (Figure 3).

The malignant biological behaviors of tumors, including proliferation [108], resistance to cell death, invasion and metastasis [109], angiogenesis, immune evasion, and tumor stem cell features, are intimately associated with the aberrant production of miRNA, lncRNA, circRNA, and piRNA in tumor cells [110]. The true nature of ncRNAs in cancer is further complicated by the fact that, in contrast to the majority of protein-coding genes, they can either promote or inhibit cancer development in a tissue and environment-dependent manner [111]. It deepens our understanding of the mechanisms underlying tumor occurrence and development. While several ncRNAs, such as piRNA, small nuclear RNA (snRNA), and small nucleolar RNA (snoRNA), have recently been demonstrated to play important regulatory roles [105], little is currently known about their roles in the development and occurrence of tumors.

### 4.2. Regulatory Mechanisms of ncRNAs in Breast Cancer

**Figure 4:**
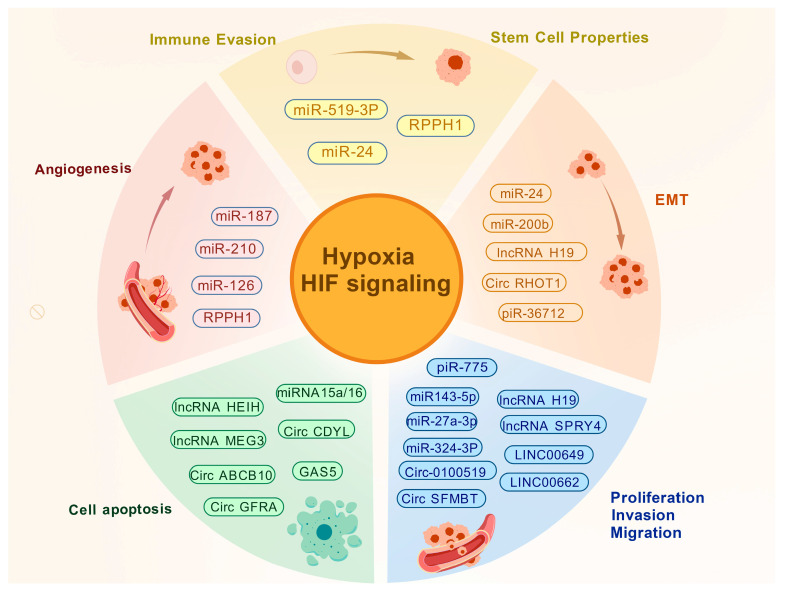
Regulatory mechanisms of ncRNAs in breast cancer. Different ncRNAs regulate hypoxia signaling pathways through various mechanisms, thereby affecting the development of breast cancer. Created with BioGDP.com accessed on 22 December 2025. Agreement number: GDP2025XJJ7VM [27].

Through multidimensional and multilayered precise regulatory mechanisms, ncRNAs serve as central molecular hubs within the gene expression regulatory network, playing key roles in major pathological processes of breast cancer, including initiation, progression, invasion, metastasis, and therapy resistance. Specifically, miRNAs primarily regulate cell division, death, and metastasis by selectively targeting and suppressing the mRNAs of oncogenes or tumor suppressor genes. Owing to the inherent stability of their circular structure, circRNAs modulate the malignant phenotype of breast cancer cells via ceRNA networks, protein-function regulation, or the encoding of small peptides. PiRNAs contribute to the cell cycle and invasive behavior of breast cancer mainly through transposon silencing and epigenetic control. lncRNAs, on the other hand, can function as ceRNAs to sequester miRNAs, recruit epigenetic modifiers to regulate gene expression, or directly interact with DNA or proteins to alter signaling pathways. Furthermore, by influencing the tumor microenvironment and mediating chemotherapy resistance, a broad range of ncRNAs profoundly shape the course and outcome of breast cancer treatment (Figure 4).

#### 4.2.1. MiRNAs in Breast Cancer

MiRNA is a short-chain non-coding RNA that is roughly 18–24 nucleotides long. It primarily controls gene expression by attaching itself to the target 3′-UTR of mRNA. In order to control transcription, certain nuclear-localized miRNAs can also directly affect a gene promoter region. Through HIF-dependent or independent mechanisms, hypoxia can control the expression of miRNAs, which impact the development of breast cancer by focusing on important molecules in the hypoxia signaling pathway.

Breast cancer cells express increased miR-27a-3p as a result of hypoxia, which may encourage malignancy. MiR-27a-3p is translocated to the nucleus by acting on the IPO8 gene and binds to the HIF-1α promoter region, attracting Argonaute 2 and RNA polymerase II to activate HIF1α transcription, which increases the activation of downstream pathways, facilitating the development, invasion, and lung metastasis of breast cancer cells. One common hypoxia-responsive miRNA that is directly controlled by HIF-1α is miR-210. It is highly expressed in breast cancer tissues and can improve tumor cells’ ability to adapt to hypoxia by blocking iron–sulfur cluster synthase activity while encouraging angiogenesis and the EMT. Through the downstream HIF-1 signaling pathway, increased miR-182 under hypoxic conditions might promote angiogenesis in breast cancers by inducing the expression of VEGF. MiR-24 has the ability to specifically control HIF-1α expression in breast cancer stem cells, which enhances the properties of cancer stem cells. Furthermore, it might hasten the malignant growth of tumors and enhance the stem cell properties and the EMT-like characteristics of breast cancer cells by increasing HIF-1α expression.

Let-7 family miRNAs functioning as tumor suppressors can target and inhibit several oncogenes to slow the growth of tumors, including E2F1, ARID3B, K-RAS, and c-Myc [112]. Tumor necrosis factor (TRAF4), which is connected to CD40 activation and B-cell receptor signaling, is affected by miR-29, which is observed in chronic lymphocytic leukemia (CLL) [113]. In CLL, downregulation of miR-29 expression raises TRAF4 expression and triggers CD40 signaling. MiR-29 expression is inhibited by activated CD40, creating a negative feedback regulation of the miR29-TRAF4-CD40 signaling axis [113]. Hypoxia can reduce the expression of miR-126, which targets VEGF and HIF-1α to prevent angiogenesis in breast cancer. Tumor vascular density and metastatic potential are increased when miR-126 expression is lost. MiR-155 can inhibit the translation of HIF-1α, lower the activity of the hypoxia signaling pathway, and limit the capacity of breast cancer cells to respond to hypoxia by directly binding to its mRNA [114]. MiR-143-5p prevents the growth and spread of cancer cells as well as the formation of tumors by post-transcriptionally inhibiting the production of HIF-1α and GLUT1 [115].

MiRNA plays a key role in regulating the biological behavior of breast cancer cells. B-ALL, or human B-cell acute lymphoblastic leukemia [116], colon cancer [117], and breast cancer [118] all have elevated expression of miR-126. B-cell leukemia is also caused by forced expression of miR-126 in mice hematopoietic stem cell progenitors. miR-155 has been identified as an oncogene in a number of malignancies, including gastric, liver, lung, colon, and breast cancers [119,120,121,122,123]. The anti-apoptotic gene B-cell-specific molony murine leukemia virus integration site 1 (BMI1) can be directly targeted by miRNA-15a/16, which is markedly downregulated in breast tissue from the standpoint of apoptosis regulation. It inhibits BMI1 inhibitory effect on the mitochondrial apoptotic pathway, causing mitochondrial-dependent apoptosis in breast cancer cells [40]. However, the apoptosis-related genes TRAIL-R2 (TNFRSF10B) and caspase-8 (CASP8] are targeted and inhibited by the oncogenic miR-519a-3p, which is highly expressed in breast cancer. It reduces the cells sensitivity to apoptotic signals, enabling breast cancer cells to evade immune surveillance and promoting tumor development [124].

Moreover, miRNAs play a crucial regulatory role in the spread of breast cancer. Of them, miR-200 is very important, and miR-200b prevents the activation of the NF-KB signaling pathway by targeting JAZF1, which lowers the expression of the EMT-related proteins on breast cancer cells and decreases their capacity for invasion and metastasis [125]. Furthermore, miR-324-3p can increase the production of lipid reactive oxygen species (ROS) by targeting glutathione peroxidase 4 (GPX4) in breast cancer cells, resulting in ferroptosis while preventing tumor cells from spreading distant [126], which offers a new potential target for the treatment of TNBC.

#### 4.2.2. LncRNA in Breast Cancer

LncRNAs are ncRNA molecules that are longer than 200 nucleotides. They can function through a variety of molecular mechanisms, including competitive ceRNA networks, RNA-binding protein interactions, and transcriptional control. Hypoxia can dramatically change the expression levels of lncRNAs in the breast cancer microenvironment, and these lncRNAs influence tumor growth by controlling HIF activity or its downstream effector molecules.

It has been established that certain lncRNAs linked to cancer promotion are responsible for the malignant development of tumors via several pathways [127]. For example, current research has established that HOTTIP is an oncogene in acute myeloid leukemia (AML), and it has been demonstrated to be significantly expressed in a variety of malignancies [128]. The epigenetically induced lncRNA1 (EPIC1) was first discovered as an oncogene in luminal B breast cancer [129]. EPIC1 has also been found to be highly expressed in a number of tumor tissues, including glioma [124], cholangiocarcinoma [130], lung cancer [131], and pancreatic cancer [132]. LncTCF7 is highly expressed in liver cancer stem cells and significantly affects the self-regulation of CSCs by attracting the Wnt signaling pathway.

Breast tissue and patient plasma exhibit elevated expression of the typical hypoxia-regulated lncRNA molecule H19, whose expression is controlled by several mechanisms, including P53 and HIF-1α. By acting as a ceRNA to adsorb miR-let-7, this molecule can increase the invasion and metastatic potential of breast cancer cells by increasing the EMT process and relaxing the inhibition of miR-let-7 on the target gene HMGA2 [133]. It lowers the methylation level of the Beclin 1 promoter region by regulating the SAHH/DNMT3B axis, activates the autophagy pathway, and eventually causes tamoxifen resistance in breast cancer cells [134]. On the other hand, H19 can promote breast cancer cell proliferation, metastasis, and cell cycle progression by expressing miR-675, interacting with MYC protein, or taking part in the ceRNA regulatory network. The dynamic fluctuations in its plasma level could be used to predict the prognosis of individuals with breast cancer and the efficacy of neoadjuvant therapy.

Hepatocellular carcinoma upregulated EZH2-associated lncRNA, or HEIH, is highly expressed in TNBC and can inhibit the JAK-STAT signaling pathway by “sponge adsorption” of miR-4458, upregulating the expression of suppressor of cytokine signaling (SOCSI), which ultimately inhibits apoptosis and promotes the proliferation of breast cancer cells [135]. RPPH1 performs both extracellular and intracellular roles, which can stimulate the PI3K/AKT pathway to increase cell stemness and invasiveness and stabilize m^6^A-modified FGFR2 mRNA by shielding IG2BP2 protein from ubiquitination destruction. RPPH1 is extracellularly packaged into exosomes and transported to endothelial cells, where it promotes angiogenesis and fosters tumor spread. Hypoxia-induced lncRNAs can improve tumor cell sensitivity to hypoxia signaling pathways by attaching to the HIF inhibitory protein VHL and blocking HIF degradation.

LncRNAs associated with tumor suppression have been shown to reverse chemotherapy resistance and decrease the malignant phenotype of breast cancer. In TNBC tissues, growth arrest-specific 5 (GAS5) is downregulated. As a tumor suppressor lncRNA, it can increase the expression of SUFU, further inhibit the activation of the Hedgehog signaling pathway, promote apoptosis of TNBC cells, reverse paclitaxel resistance, and relieve the inhibition of the tumor suppressor gene SUFU by miR-378a-5p through “sponge adsorption” of miR-378a-5p [136]. The lowly expressed lncRNA MEG3 can cause breast cancer cells to undergo apoptosis and increase the chemotherapeutic agent cisplatin’s ability to kill TNBC via triggering the NLRP3 inflammasome/caspase-1/GSDMD pathway.

Multiple lncRNAs are important in the invasion, metastasis, and poor prognosis of breast cancer [137]. The lncRNAs SPRY4 intronic transcript (SPRY4-IT1) by controlling the expression of HIF-1α [138], which is abundantly expressed in breast cancer tissues, can encourage cancer cell metastasis. The poor prognosis for breast cancer is linked to increased expression of the lncRNAs MIR210HG, which directly binds to the 5′UTR of HIF-1α to stabilize its production. LINC00649 can encourage the growth and spread of breast cancer tumors by maintaining HIF-1α expression.

Several lncRNAs target and regulate hypoxia-related pathways to stimulate tumor growth during the invasion and proliferation of breast cancer cells: miR-597-3p is eliminated from the hypoxia-inducible factor 2 (EglN2) of the egl-9 family [139], the inhibition of EglN2 is lessened, and LINC00662 stimulates the growth and invasion of cancer cells. In addition to being engaged in the malignant expression regulation of breast cancer cells, CHRTI expression is elevated in TNBC tissues. Versican (VCAN)-AS1 is an endogenous RNA that targets signal transducer and activator of STAT3 to inhibit miR-106a-5p and promotes the growth of cancer cells by blocking the STAT3/HIF-1α axis [140].

#### 4.2.3. CircRNA in Breast Cancer

Reverse splicing of precursor mRNA results in circRNA, a closed circular molecule. It has great tissue specificity, robust nuclease resistance, and structural stability. It mostly binds to miRNA or RNA-binding proteins (RBP) to perform regulatory actions. According to recent research, a number of circRNAs are selectively increased in the hypoxic environment of breast cancer and play a significant role in the formation and occurrence of tumors by changing the tumor microenvironment or controlling the tumor malignant phenotype [141].

CircRNA can be an oncogene or a tumor suppressor. For example, TNBC, bladder cancer, and colon cancer all have downregulated circCDYL. Its overexpression can stop the growth of tumor cells and encourage apoptosis in breast cancer cells. CircCCAC1, or cholangiocarcinoma-associated circular RNA1, is significantly expressed in endothelial cells generated from cholangiocarcinoma and has been identified as an oncogene [142]. In hypoxia-treated breast cancer MCF-7 cells, the expression of the hypoxia-responsive circSFMBT2 is markedly elevated. Functional studies have demonstrated the tumor suppressor activity of cicrSFMBT by confirming that its overexpression can prevent the proliferation, migration, invasion, and the EMT of breast cancer cells. Although no interaction with miRNA was discovered, RNA pulldown tests have shown its binding to RBP PABPC1, indicating that it may carry out its activity by binding to RBP.

Certain circRNAs contribute to the invasion and metastasis of breast cancer and are linked to a poor prognosis for patients by controlling the tumor microenvironment. In breast cancer tissues, circ0100519 is expressed and enriched. To increase its transcription, HIF-1α can function as an upstream regulatory factor. Circ0100519 can be encapsulated in exosomes and transported to tumor-associated macrophages by serving as a molecular scaffold to enhance the interaction between the deubiquitinating enzyme USP7 and nuclear factor NRF2 [143], which results in NRF2 deubiquitination modification mediated by USP7, in turn stimulating M2-type macrophage polarization and eventually accelerating breast cancer invasion and metastasis. The method also reveals the importance of circRNAs in the alteration of the hypoxia-regulated tumor microenvironment [144].

CircRNA is important for controlling treatment resistance in breast cancer. By “sponge adsorption” of let-7a-5p, circABCB10, which is highly expressed in paclitaxel-resistant breast cancer tissues, can increase the expression of the Dual-specificity phosphatase 7 (DUSP) gene, prevent the activation of the MAPK signaling pathway, decrease breast cancer cell apoptosis, and cause paclitaxel resistance [145]. Conversely, circBGN is highly expressed in trastuzumab-resistant HER-2 positive breast cancer cell lines and inhibits ferroptosis of breast cancer cells by regulating the expression of ovarian tumor protease domain-containing deubiquitinase 1 (OTUB) and solute carrier family 7 member 11 [SLC7A11], which reduces the tumor cells’ sensitivity to trastuzumab [146].

CircRNAs have pro-metastatic effects during the control of breast cancer metastasis by focusing on and controlling important signaling pathways. RHOT1 facilitates the EMT process, invasion, and metastasis of breast cancer cells by “sponge adsorbing” miR-106-5p, upregulating the expression of signal transducer and activator of transcription 3 (STAT3), and initiating the STAT3 signaling pathway. It prevents cells from undergoing ferroptosis, offering a fresh target for the treatment of breast cancer metastases [147]. CircGFRA is abundantly expressed in HER-2 positive breast cancer, prevents apoptosis, and promotes breast cancer cell metastasis by adsorbing miR-1228 and upregulating the expression of apoptosis-inducing factor mitochondria-associated 2 (AIFM2), which is related to poor prognosis for breast cancer.

#### 4.2.4. PiRNA in Breast Cancer

The piRNA is roughly 24–32 nucleotides long and interacts with the Piwi protein family. It is distinguished by a variety of biological roles, tissue-specific enrichment, and highly conserved sequences. It primarily works by controlling mRNA stability, transposon suppression, and transcriptional silencing. It has been reported that the hypoxic microenvironment of breast cancer specifically modifies several piRNA expression patterns [148]. They play a significant role in the onset, progression, and metastasis of breast cancer by controlling the malignant biological characteristics of tumor cells or influencing the interaction between tumor cells and the milieu.

Transposons, also referred to as “jumping genes,” are DNA sequences in the genome that can travel on their own. They play a significant role in the malignant growth of cancer cells and can cause genetic abnormalities and rearrangements that upset the equilibrium between cell proliferation and apoptosis. PiRNA accurately identifies and attaches to the RNA transcripts or DNA sequences of transposons in breast cancer cells by creating a piRNA–Piwi complex with Piwi proteins [144]. It prevents genomic abnormalities brought on by aberrant jumping by directly silencing transposon activity by cutting transposon RNA and inducing transposon DNA methylation [149]. The loss of cell cycle control in breast cancer cells (e.g., abnormal G1/S checkpoint, unrestrained cell proliferation) is often associated with aberrant transposon activation. PiRNA can correct aberrant cell cycle progression and restore genomic integrity by silencing transposons. Uncontrolled transposons will accelerate the cell cycle and proliferation of breast cancer cells if piRNA expression is downregulated. Moreover, normal or upregulated piRNA expression can limit transposon activity and postpone or stop the aberrant proliferation of cancer cells. Epigenetic regulation is the control of gene expression by mechanisms such as DNA methylation, histone modification, and chromatin remodeling without altering the DNA sequence. PiRNA can affect the ability of breast cancer cells to invade through epigenetic processes [150].

PiRNA can be an oncogene or a tumor suppressor. For example, TNBC tissue exhibits markedly reduced expression of piR-775 [151]. Its overexpression can reduce the growth, migration, and invasion of breast cancer cells and increase the sensitivity of tumor cells to PARP inhibitors by targeting and controlling the expression of DNA damage repair genes XRCC2/BARD1 and cell cycle-related gene TPX2 [152]. Particularly highly expressed in breast stem cells, piR-932 functions as an oncogene by encouraging the methylation of CpG islands in the Latexin gene promoter region. It increases the ability of breast stem cells to self-renew and spread, which is closely linked to the malignant development of tumors [153]. Under hypoxic therapy, the expression level of the hypoxia-responsive piRNA PiR-36712 is markedly elevated in breast cancer cells. Through competitive binding to miR-7/miR-324, piR-36712 overexpression can control the expression of SEPW1 by activating the P53 signaling pathway, downregulating the transcription component Slug, and upregulating the epithelial marker E-cadherin, according to functional tests. In the end, it clearly has a tumor suppressor effect by preventing the growth, invasion, and the EMT of breast cancer cells [154].

### 4.3. Oxygen-Driven ncRNAs in Breast Cancer

In breast cancer, aberrant expression of oncogenes like Ras and Myc, along with aberrant activation of PI3K/Akt and MAPK signaling pathways, can further increase the stability and transcriptional activity of HIF-1α through phosphorylation modification, creating a superimposed effect of hypoxia adaptation. The aggressiveness and poor prognosis of breast cancer tumors are strongly associated with aberrant activation of hypoxia signaling pathways. Important molecules like HIF-1α, H19, and EZH2 have emerged as possible therapeutic targets in this process. Notably, ncRNA networks allow the hypoxic molecular signaling pathways in breast cancer to have regulatory effects. In particular, hypoxia can increase the expression of lncRNA H19, which functions as a ceRNA to bind miRNA let-7 with EZH2 mRNA in a competitive manner, so indirectly increasing EZH2 expression and encouraging the invasion and metastasis of breast cancer cells [40].

Hypoxic circumstances can affect the expression of a variety of ncRNAs in addition to controlling a huge number of protein-coding genes through both HIF-dependent and independent signaling pathways [155]. Transcriptional and post-transcriptional regulation at different levels, including editing, modification, maturation, stability, localization, and transport, is involved in the underlying molecular mechanisms [156,157,158]. For instance, modifications in the editing patterns of miRNAs can target many mRNAs simultaneously, particularly when miRNAs are edited, and can modify the entire transcriptome. According to recent research, hypoxia is a crucial external stimulation that can change the cell whole transcriptome profile by editing in the miRNA seed regions [159]. Hypoxia may also have an impact on the conversion of miRNA transcripts into mature miRNAs in breast cancer by blocking the crucial enzyme Dicer involved in the synthesis of miRNAs [160,161]. When EGFR binds to Ago2 and phosphorylates it at Tyr393, the connection between Ago2 and Dicer is broken in a hypoxic environment [162], impairing miRNA maturation. It promotes the invasion and spread of breast cancer by enabling several oncogenes to evade miRNA repression. Moreover, hypoxia carefully controls the expression of a number of ncRNAs, such as miRNAs, lncRNAs, circRNAs, and snRNAs, which accelerates the development of cancer [158].

#### 4.3.1. HIFs Regulate the Expression of ncRNA

HIFs, the fundamental transcriptional regulatory factor for cells to adapt to hypoxic microenvironments, can precisely regulate the expression of lncRNAs, miRNAs, and other ncRNAs by either directly attaching to HREs in target gene promoter regions or indirectly regulating signaling pathways. The “HIF-ncRNA” regulatory network significantly affects important biological processes such as tumor occurrence and development, metabolic reprogramming, and treatment response. Members of the HIF family have direct control over the transcriptional activation of lncRNAs. HIF-1α, HIF-2α, and HIF-1β may all bind to the promoter region of HILRNA68 in hypoxic liver cancer cells, raising its expression by about ten times. Consequently, HILRNA68 can increase HIF-1α transcriptional activity, creating a positive feedback loop that greatly increases tumor cell invasion and proliferation. Similarly, HIF-2α can increase the invasive and metastatic potential of liver cancer cells by enhancing the EMT process and increasing the expression of lncRNA NEAT1 [163]. HIF-1α can cause the expression of its antisense RNA, HIF1A-AS2, in clear cell renal cell carcinoma. HIF1A-AS2 then directly binds to the Gli1 protein to cooperatively regulate the expression of HIF-1α itself, hastening the growth of the tumor. Furthermore, HIF-1α can trigger the synthesis of lincRNA-p21, which stabilizes the protein level of HIF-1α and increases the glycolytic metabolic activity of tumor cells by preventing the ubiquitination and destruction of HIF-1α by interfering with the interaction between VHL and HIF-1α. Tamoxifen treatment can cause lncRNA UCA1 expression to be upregulated in breast cancer cell lines in a way that is dependent on HIF-1α [163].

#### 4.3.2. NcRNA Inversely Regulates the Activity or Stability of HIFs

LncRNAs can carry out their tasks by attaching to HIF proteins or their gene regulatory areas. HIFCAR can engage HIF-1α and P300 to form a transcriptional complex that promotes target gene transcription and tumor growth by directly binding to the promoter region of HIF-1α target genes in oral cancer. However, lncRNA competitively binds to YB-1 protein and blocks it from attaching to the 5′UTR region of HIF-1α mRNA through its highly specific YB-1 binding motif, leading to HITT suppressing the translation process of HIF-1α. HIF can directly transcribe and activate the antisense transcript of HIF-1α by altering the transcriptional status of the HIF-1α gene and creating a self-feedback regulatory loop, which contributes to the control of HIF-1α expression.

One significant method by which ncRNA indirectly controls HIF is through the ceRNA pathway. The lncRNA ASLNC12089 functions as a ceRNA in non-small cell lung cancer, competing with HIF-1α mRNAs and the HIF-1α chaperone protein (HIF1AN) for binding to miRNAs via common miRNA response elements (MREs). It controls the mRNA stability under various oxygen circumstances, preserving the relative balance of HIF-1α/HIF1AN and preventing the growth of tumors. Through ncRNA, the HIF pathway itself can create an indirect feedback control. For example, HIF-1α can trigger the expression of miR-205, which can directly target and degrade lncRNA HITT. It creates a feedback loop of HITT-HIF-1α-miR-205 for self-regulation, thereby indirectly regulating HIF-1α expression and activity. A variety of ncRNAs (miRNAs, lncRNAs, etc.) that are directly transcribed by HIF can function as effector molecules, mediating the biological functions of HIF by influencing downstream pathways such as cell metabolism and RNA processing, and are involved in the development of the tumor’s hypoxic phenotype.

In hypoxic cancer cells, HIF-1α and H19 expression are significantly correlated among lncRNAs. H19 may be involved in the regulation of HIF-1α by acting as a ceRNA and sequestering many miRNAs implicated in HIF-1α control [164]. Elevated H19 levels cause HIF-1α expression to rise and further activate its downstream effectors. The significant involvement of H19 in the regulation of HIF-1α was subsequently validated by Corrado et al.’s findings: HITAIR functions as a ceRNA in the regulation of HIF-1α [165,166,167]. Long intergenic non-coding RNA kinase activator (LINK-A) was found to regulate the activation of HIF-1α under normoxic conditions in TNBC. RNA-p21 may contribute to the control of HIF-1α and enhance its stability by competitively binding to the VHL protein [124]. HOTAIR can regulate HIF-1α expression in renal cell carcinoma by sequestering the tumor suppressor miR-127.

## 5. The Role of Hypoxia-Responsive ncRNAs and HIF Signaling Pathway in Clinical Breast Cancer

LncRNAs are present in many cellular processes in animals because of their broad participation in post-transcriptional control, epigenetic regulation, and even signal transmission. LncRNAs are dysregulated in many cancers, according to multiple studies [168], and their expression patterns are linked to clinical aspects, suggesting that they could be used for early diagnosis and prognosis [169]. Cell proliferation is one of the many malignant biological activities, and lncRNAs can regulate [170,171] invasion [172], migration [171,172,173], apoptosis [174], EMT [175], maintenance of stem cell phenotypes, responses to chemotherapy drugs [176,177], and core cancer characteristics. While the regulation mechanisms of non-cell autonomous signals mediated by the tumor microenvironment are still unknown, the majority of related research now focuses on the effect of lncRNAs on cell autonomy [178]. Phenotypic plasticity and non-mutational epigenetic reprogramming (NMER), two emerging characteristics of cancer, have been linked to these ncRNAs [179]. Numerous investigations conducted recently have demonstrated that hypoxia controls the expression of lncRNAs [157,180,181]. In the HIF-dependent regulatory mode, HIF-2α can directly transcribe and activate lncRNA NEAT1, cause the nucleus to form paraspeckle structures, and decrease the protein expression of RNA transcripts like F11r by keeping them. In the end, this promotes cell proliferation, increases the capacity for clonal formation, and prevents apoptosis. The poor prognosis for patients is strongly linked to increased expression of NEAT1 in breast tissue. Similarly, HIF-2α can selectively upregulate lncRNA RAB11B-AS1, recruit RNA polymerase II to increase the expression of angiogenic factors including VEGFA and ANGPTL4, and encourage the creation of endothelial cell tubes and distant metastasis of breast cancer.

Through scaffold roles, lncRNAs can regulate not just the HIF signaling pathway but also the stability and activity of HIF-1α. LINC00115 functions as a scaffold molecule to control the SETDB1/PLK3/HIF1α signaling pathway in paclitaxel-resistant breast cancer stem cells. It influences the phosphorylation and degradation of HIF1α, improving the stemness, chemoresistance, and metastatic potential of tumor stem cells [182]. Research has demonstrated that lncRNAs such RAB11B-AS1 and NEAT1 are linked to a poor prognosis in breast cancer, making them possible prognostic markers [183]. A novel strategy for overcoming treatment resistance in breast cancer is to target LINC00115 or SETDB1 to modulate HIF-1α signaling. In this section, we provide an overview and explanation of commonly used ncRNA therapeutics, along with their current clinical application status. For detailed information, please refer to Table 2.

## 6. Conclusions

The hypoxic microenvironment, a common phenomenon in the development of breast cancer, stimulates fundamental pathways like angiogenesis and metabolic reprogramming, encouraging tumor growth, metastasis, and treatment resistance by stabilizing HIFs. There are several different kinds of ncRNAs, including piRNA, lncRNA, circRNA, and miRNA. Through various molecular pathways, these molecules take part in controlling the progression of various malignancies. The relationship between ncRNAs and hypoxic molecular signaling pathways is increasingly more understood. A crucial foundation for molecular typing and prognostic evaluation of breast cancer is provided by the fact that different ncRNAs have distinct modes of action in various molecular subtypes of the disease, and their aberrant expression is strongly associated with the malignant phenotype and poor prognosis of tumors. Nonetheless, there are still a lot of unidentified ncRNAs, including novel ncRNA classes with specific roles that have not been well investigated, particularly their functions and mechanisms in breast cancer. There is still a need to investigate the clinical utility of known ncRNAs. Their enormous potential for drug development must be confirmed by preclinical and clinical trials for safety and efficacy, particularly for hard-to-treat subtypes like TNBC, which is anticipated to break through current treatment bottlenecks and offer fresh approaches for the development of combined treatment strategies, ultimately leading to a significant advancement in the diagnosis and treatment of breast cancer. There is still a need to experiment with more cutting-edge therapeutic approaches in the future, such as integrating ncRNAs with immunotherapy or other therapeutic approaches, which could lead to unanticipated advancements in clinical treatment.

## Figures and Tables

**Table 1:** NcRNAs in breast cancer subtypes.

Breast Cancer Subtypes		NcRNAs	Refs
Luminal A	ER (+)	lncRNA PVT1, lncRNA LINC01016, lncRNA LINC00160, lncRNA DSCAM-AS1, lncRNA HOTAIRM1, AP001528.2, LINC00968, LINC02202, TRHDE-AS1, LINC01140	[82,83,84]
	PR (+)	lncRNA EGOT, lncRNA CCAT1, PDCD4-AS1, MEG3, AP001528.2, LINC00968, LINC02202, TRHDE-AS1, LINC01140, AL354707.1, AC097534.1, MIR222HG, AL662844.4, miR-29a, miR-181a, miR-223, miR-652	[84,85,86]
	HER-2 (−)	AP001528.2, LINC00968, LINC02202, TRHDE-AS1, LINC01140, AL354707.1, AC097534.1, MIR222HG, AL662844.4, lncRNA CCAT1, PDCD4-AS1, MEG3, lncRNA ENST00000460164, miR-30	[84,85]
	Low expression of Ki-67	AP001528.2, LINC00968, LINC02202, TRHDE-AS1, LINC01140, AL354707.1, AC097534.1, MIR222HG, AL662844.4, lncRNA CCAT1, PDCD4-AS1, MEG3, lncRNA ENST00000460164, lncRNALinc00707, miR-30	[84,85]
Lumina B	ER (+)	DSCAM-AS1, MIAT, MALAT1, MIAT, WT1-AS, PVT1, EPB41L4A-AS1, FGD5-AS1	[87]
	PR (+)	lncRNA01089-209, NCALD, EGOT, MALAT1, MIAT, WT1-AS, miR-30a-3p	[88,89]
	HER-2 (−)	lncRNA01089-209, HAGLR, miR-548b-5p, miR-451a, U6 snRNA	[90]
	High expression of Ki-67	BC069792, miR-548b-5p, miR-4739, miR-658	[90]
HER-2 overexpression	HER-2 (+)	AC092718.4, AFAP1-AS1, ES3, HOTAIR, snaR, LINC00636, LINC01405, ADARB2-AS1, ST8SIA6-AS1, LINC00511, DPP10-AS1, linc-SLC39A10-10, linc-GJA1-2, linc-STARD6-2, miR-135a-5p, miR-29b	[91,92,93,94]
	ER (−)	LncRNA ES3, lncRNA lnc-ATB, lncRNA SNHG14, miR-125, miR-331-3P	[91,92]
	PR (−)	AFAP1-AS1, PVT1, LOC145837, FLJ40504, FLJ45983, snaR, KCNQ1OT1, NEAT1, ES3, miR-4728-3p, miR-146a-5p, miR-181d, miR-195-5p, circHER2, circPVT1	[92,93,94,95]
	High expression of Ki-67	circCDYL2, MVIH	
Triple-negative	ER (−)	RNA CDKN2B-AS1, AK124454, LINC00461, AFAP1-AS1, FAISL	[96,97]
	PR (−)		
	HER-2 (−)		
	High expression of Ki-67	LncRNA-P21, miR-17-3p, FAISL, LINC00571	

**Table 2:** Common ncRNA drugs and their clinical application.

Drugs	Targeting	Indications	In Clinic	Refs
tRNA-Asp-GTC-3′tDR	unpublished	Kidney diseases (renal ischemia-reperfusion injury)	In the preclinical research stage	
GTX-102	unpublished	Angelman syndrome	In the early stage of clinical research	[184]
Donidalorsen	unpublished	Hereditary angioedema	The FDA has accepted the marketing application, and the PDUFA date is set for 21 August 2025.	[185,186]
miR-21 inhibitor	miR-21	Lung cancer and other cancers	Animal experiments have shown anticancer effects, and it is currently in the preclinical research stage.	[187,188]
ARO-AAT	SERPINA mRNA	Rare hereditary liver diseases related to alpha-1 antitrypsin deficiency (AATD)	The clinical Phase II trial was completed in 2020.	[189]
Elebsiran (VIR-2218)	HBV	Chronic hepatitis B	Phase I clinical trial	[190]
IONIS-HTTRX	HTT mRNA	Huntington’s disease	Phase III clinical trial	[191,192]
SPR-9001	dystrophin mRNA	Duchenne muscular dystrophy (DMD)	It was approved for marketing by the FDA in 2023.	[193]
Fitusiran	Antithrombin (AT) mRNA	Hemophilia A and hemophilia B	It was approved for marketing in the European Union in 2020.	[194,195]
Amvuttra	TTR mRNA	Hereditary transthyretin-mediated amyloidosis (hATTR)	It received FDA approval in 2022.	[196]
QP1-1007	Capase 2	Acute non-arteritic anterior ischemic optic neuropathy	In the preclinical research stage	[197,198]
EYE001	VEGF	Age-related macular degeneration	In the preclinical research stage	[199]
Tivanisiran	TLR7	Xerophthalmia	It was approved for marketing by the FDA in 2023.	[200]
Givosiran	ALAS1 mRNA	Acute Intermittent Porphyria (AIP)	It was approved for marketing by the FDA in 2019.	[201,202,203]
Patisiran	TTR mRNA	Hereditary transthyretin-mediated amyloidosis (hATTR)	It was approved in 2018 and is the first siRNA drug to be marketed.	[204,205,206,207]
lnclisiran	PCSK9	Patients with high cardiovascular risk	It was approved for marketing in the European Union in December 2020.	[208]
Bepirovirsen	HBV RNA	Chronic hepatitis B	Phase III clinical trials are currently underway.	[182,209]
Res-010	MiR-22	Obesity and fatty liver disease	Phase I clinical trials are currently underway.	[210]
SiG12D-LODER	KRASG12D	Locally advanced pancreatic cancer	Phase II clinical trials are currently underway.	[211,212]
TKM-080301	PLK1 mRNA	Advanced hepatocellular carcinoma	Phase II clinical trials have been completed, and development has been halted.	[213]
Atu027	PKN3	Advanced solid tumors, locally advanced or metastatic pancreatic cancer	In the clinical research stage	[214]
QR-110	CEP290	Leber congenital amaurosis	In the clinical research stage	[215,216]

## Data Availability

No new data were created or analyzed in this study.
